# Supplementary material for: The multidomain architecture of a bacteriophage endolysin enables intramolecular synergism and regulation of bacterial lysis
Source: J Biol Chem. 2021 Apr 8;296:100639. doi: 10.1016/j.jbc.2021.100639 (PMC8144678; doi:10.1016/j.jbc.2021.100639)
Supplement: Suppplemental Figures S1–S5 and Tables S1–S5 [file mmc3.docx]

**Supplementary Table 1:** Bacterial strains and their origins, plasmids and nucleotides

| **Bacterial strains** | **Characteristics** | **Origin** | |
| --- | --- | --- | --- |
|  |  |  | |
| ***E. coli*** |  |  | |
| BL21(DE3)pLysS | F^-^ *ompT hsdSB* (r_B_ ^-^ m_B_^-^) *dcm*^+^*gal* (DE3) pLysS (Cam^r^ ) | Stratagene | |
| BL21/pPlySK1249^28a^ | *E. coli* BL21 (DE3) (pLysS) transformed with pPlySK1249^28a^ | This study | |
| BL21/pAmi^28a^ | *E. coli* BL21 (DE3) (pLysS) transformed with pAmi^28a^ | This study | |
| BL21/pAmi_LysM^28a^ | *E. coli* BL21 (DE3) (pLysS) transformed with pAmi_LysM^28a^ | This study | |
| BL21/pLysM_CHAP^28a^ | *E. coli* BL21 (DE3) (pLysS) transformed with pLysM_CHAP^28a^ | This study | |
|  |  |  | |
| **Streptococci** |  |  | |
| *S. dysgalactiae* SK1249 | Human, haemoculture | K. Mogens | |
| *S. agalactiae* FLS-S3-026 | Bovine | M. Stanhope | |
| *S. agalactiae* 17-2167 | Human, endocarditis | CHUV^b^ | |
| *S. agalactiae* 532 | Human | G. Jordan | |
| *S. agalactiae* GF | Human, haemoculture | F. Gilliand | |
| *S. pyogenes* ATCC 19615 | Human, sore throat | R.G. Wittler | |
| *S. gordonii* DL1 | Human | H.F. Jenkinson | |
| *S. mutans* ATCC 25175 | Human, carious dentine | DSMZ^c^ | |
| *S. suis #*19 | Porcine | M. Haenni | |
| *S. uberis* ATCC 700704 | Bovine | ATCC^d^ | |
| *S. pneumonia* D39 | Human | J. McCuller | |
| **Enterococci** |  |  | |
| *E. faecalis* ATCC 29212 | Human, urine | Micro-Media Systems, Inc | |
| *E. faecium* D344 | Human | J. Entenza | |
|  |  |  | |
| **Plasmids** | **Characteristics^a^** | **Origin** | |
|  |  |  | |
| pET-28a | Expression vector; Kan^r^ | Novagen | |
| pPlySK1249^28a^ | pET-28a carrying *PlySK1249* | This study | |
| pAmi^28a^ | pET-28a carrying *Ami* | This study | |
| pAmi_LysM^28a^ | pET-28a carrying *Ami_LysM* | This study | |
| pLysM_CHAP^28a^ | pET-28a carrying *LysM_CHAP* | This study | |
|  |  |  | |
| **Oligonucleotides** | **Sequence 5’ → 3’, restriction enzyme^e^** | **Origin** | |
|  |  |  | |
| plySK28aNcoIFw | GCATGCCATGGGAAAACATCTAGTGATTTGTGGACATGGGCAAGGACG | This study | |
| plySK28aXhoIRv | GCCGCTCGAGTGAAATTCTAAACCAACCTACAACTTTTCCAAGTTTAACTGTTCCAG | | This study |
| Ami28aXhoIRv | GCCGCTCGAGTGTTATTGCGGACACAAGACCTTTCG | | This study |
| LysMCHAP28aNcoIFw | GCATGCCATGGAATCATGGGTGCTGGAGCAGGATGTAGAAGGAAC | | This study |
| T7 | TAATACGACTCACTATAGGG | | Novagen |
| T7 terminator | GCTAGTTATTGCTCAGCGG | | Novagen |
| ^a^ Abbreviations: Cam^r^, chloramphenicol resistance; Kan^r^, kanamycin resistance ^b^ Centre hospitalier universitaire vaudois (CHUV, Switzerland) ^c^ Deutsche Sammlung von Mikroorganismen und Zellkulturen (DSMZ, Leibniz-Institut, Germany) ^d^ American type culture collection (ATCC, Manassas, Virginia, USA) ^e^ Restriction sites are underlined. | | | |

|  |  |  |
| --- | --- | --- |
|  |  |  |
|  |  |  |

**Supplementary Table 2.** Park Johnson and Ghuysen assays for differentiation of glycosidase and amidase/endopeptidase activity of different PGHs against purified *S. aureus* cell walls

|  | **Reducing activity ^a^  [μg glucose]** | | | **Free NH_2_ groups ^b^ [mM]** | | |
| --- | --- | --- | --- | --- | --- | --- |
| PlySK1249 | 1.9 | ± | 25.9 | 8.1 | ± | 2.7 |
| Ami | 13.3 | ± | 8.0 | 11.4 | ± | 0.3 |
| Ami_LysM | 3.0 | ± | 26.0 | 3.0 | ± | 1.2 |
| CHAP | 6.3 | ± | 25.0 | 5.7 | ± | 2.6 |
| Mutanolysin | 438.4 | ± | 111.1 | 0.1 | ± | 0.2 |

^a^ Quantification of reducing groups was performed using the Park-Johnson assay. Reducing activity is expressed as μg glucose equivalents released in the incubation mixture.

^b^ The concentration of free amino groups was determined with the Ghuysen assay.

**Supplementary Table 3.** Amino acid sequencing of bands 1 and 2 that were extracted from the gel presented in Figure 4.A. Peptides were quantified based on signal intensity (precursor mass intensity). Relative comparison of those intensities according to the peptide position in the PlySK1249 sequence allows the estimation of which parts of the protein are present in the different bands and to calculate their MW.

|  | **band 1: MSC_F005002** | **band 2: MSC_F005003** |
| --- | --- | --- |
| **Score** | 7748 | 6882 |
| **# MS/MS Spectra** | 186 | 171 |
| **# Peptides** | 97 | 84 |
| **% Coverage** | 82 | 86 |
| **Part of the sequence covered (based on relative peptide intensities)** | 1-204 & 318-478 | 1-175 |
| **Calculated MW (Da)** | 22,300 & 18,400 | 19 400 |

| **Peptide position** | **Peptide Sequence** | **Start Pos** | **End Pos** | **Spectrum Id** | **Prec Rt** | **Prec m/z** | **Prec Charge** | **Mascot Score** | **Mass Diff [ppm]** | **band 1: MSC_F005002 Rt** | **band 2: MSC_F005003 Rt** | **band 1: MSC_F005002 Intensity** | **band 2: MSC_F005003 Intensity** |
| --- | --- | --- | --- | --- | --- | --- | --- | --- | --- | --- | --- | --- | --- |
| 4-14 | K.HLVIC{Carbamidomethyl}GHGQGR.T | 4 | 14 | 4390 | 27.43 | 411.880 | 3 | 45.8 | -0.47 | 27.44 | 27.49 | 2.61E+08 | 1.42E+08 |
| 15-25 | R.TTYDPGAVNAK.L | 15 | 25 | 4647 | 28.00 | 568.782 | 2 | 58.2 | -1.07 | 28.06 | 28.09 | 1.21E+09 | 9.27E+08 |
| 44-61 | K.YSGQQIDFITEQNVYDYR.S | 44 | 61 | 7643 | 41.12 | 747.014 | 3 | 89.7 | -0.61 | 40.73 | 40.85 | 5.43E+08 | 4.25E+08 |
| 69-86 | K.GYDSITELHFNAFNGSAK.G | 69 | 86 | 6914 | 38.04 | 657.647 | 3 | 60.8 | 0.75 | 38.18 | 38.23 | 1.65E+08 | 1.54E+08 |
| 87-100 | K.GTEVLIQSSLEADK.E | 87 | 100 | 6657 | 35.58 | 745.392 | 2 | 97.8 | 1.71 | 35.61 | 35.71 | 2.75E+06 | 1.57E+06 |
| 120-133 | K.KVDWLYNANQAASR.G | 120 | 133 | 5571 | 32.72 | 545.947 | 3 | 71.7 | 1.27 | 32.83 | 32.89 | 7.19E+08 | 4.42E+08 |
| 139-157 | R.LVEIAFIDNEQDMAIFETK.K | 139 | 157 | 9720 | 48.01 | 742.705 | 3 | 92.1 | -0.11 | 48.18 | 48.21 | 1.68E+07 | 1.10E+07 |
| 164-175 | K.GLVSAITGVEVK.T | 164 | 175 | 7270 | 37.80 | 586.848 | 2 | 67.8 | 0.39 | 37.88 | 37.95 | 4.71E+08 | 6.96E+08 |
| 176-194 | K.TIVPSTPSSTVGSSGTPSK.P | 176 | 194 | 5133 | 30.45 | 895.463 | 2 | 90.1 | 1.71 | 30.63 | 30.59 | 1.32E+07 | 9.22E+05 |
| 176-204 | K.TIVPSTPSSTVGSSGTPSKPIYLVGDSLR.V | 176 | 204 | 7192 | 37.53 | 726.640 | 4 | 61.9 | 1.01 | 37.6 | 37.69 | 1.31E+08 | 1.39E+07 |
| 205-217 | R.VLPHATHYQTGQK.I | 205 | 217 | 4397 | 27.37 | 493.929 | 3 | 36.1 | 2.39 | 27.36 | 27.42 | 1.03E+08 | 1.52E+08 |
| 234-242 | K.NVHQSNSLR.A | 234 | 242 | 4222 | 26.71 | 527.773 | 2 | 31.9 | 0.34 | 26.77 | 26.80 | 8.89E+04 | 2.73E+05 |
| 243-250 | R.AYLLDGIK.S | 243 | 250 | 6880 | 36.37 | 446.761 | 2 | 34.7 | 0.01 | 36.43 | 36.54 | 5.36E+07 | 9.28E+07 |
| 251-263 | K.SWVLEQDVEGTTK.G | 251 | 263 | 6482 | 36.24 | 746.371 | 2 | 71.3 | 1.39 | 36.81 | 36.88 | 3.35E+07 | 7.46E+07 |
| 264-274 | K.GHSEQTYQAQK.G | 264 | 274 | 4190 | 26.56 | 426.202 | 3 | 32.0 | -0.97 | 26.56 | 26.59 | 6.29E+04 | 2.21E+05 |
| 275-283 | K.GDTYYGIAR.K | 275 | 283 | 5266 | 31.10 | 508.246 | 2 | 41.4 | 0.46 | 31.18 | 31.19 | 2.35E+07 | 6.81E+07 |
| 306-317 | R.VGQTLKVNAASR.I | 306 | 317 | 4479 | 27.74 | 415.242 | 3 | 58.7 | -0.27 | 27.73 | 27.76 | 1.01E+06 | 2.27E+06 |
| 318-329 | R.ITTAIPTSVASR.V | 318 | 329 | 5543 | 32.24 | 608.848 | 2 | 68.4 | -1.34 | 32.34 | 32.39 | 1.65E+09 | 1.47E+08 |
| 342-359 | K.VTVPSNPYGGQC{Carbamidomethyl}VALVDK.I | 342 | 359 | 6809 | 36.12 | 635.325 | 3 | 86.0 | 1.91 | 36.00 | 36.13 | 1.16E+08 | 2.56E+07 |
| 360-367 | K.IVQELTDK.N | 360 | 367 | 4633 | 28.32 | 473.266 | 2 | 36.2 | 0.60 | 28.70 | 28.73 | 7.14E+08 | 2.18E+08 |
| 368-379 | K.NMSYTNAIDC{Carbamidomethyl}LK.K | 368 | 379 | 6721 | 35.83 | 715.328 | 2 | 74.3 | 2.46 | 35.86 | 35.95 | 4.14E+07 | 7.62E+06 |
| 383-398 | K.SNGFQVIYDAWGVNPK.A | 383 | 398 | 8812 | 44.09 | 897.945 | 2 | 81.4 | 1.42 | 44.08 | 44.10 | 4.87E+07 | 1.22E+07 |
| 426-442 | K.SIDGVEQNIDGYSDHNK.N | 426 | 442 | 5281 | 31.18 | 630.954 | 3 | 69.5 | 0.04 | 31.19 | 31.22 | 5.77E+08 | 1.62E+08 |
| 443-458 | K.NGINDQLEIGGGGITR.R | 443 | 458 | 6329 | 35.59 | 807.416 | 2 | 88.5 | 0.56 | 35.69 | 35.80 | 2.50E+08 | 5.09E+07 |
| 463-478 | R.QWMADGSLYDSTGTVK.L | 463 | 478 | 7063 | 37.05 | 879.905 | 2 | 102.3 | 1.00 | 37.12 | 37.20 | 1.34E+08 | 2.96E+07 |

**Supplementary Table 4.** Relative abundances after LC-MS analysis of the protease content present in the fractions B4, B6, B8 and B10 (Figure 5.C). The number of distinct peptides and coverage assigned for each protein are indicated in brackets. A list of all peptide sequences identified, precursor charge and m/z for each assignment, observed modification and identification score can be found in Supplemental Data 1.

| **Identified Proteins** | **Accession Number** | **Alternate ID** | **Molecular Weight** | **Frct B4** | **Frct B6** | **Frct B8** | **Frct B10** |
| --- | --- | --- | --- | --- | --- | --- | --- |
| Peptidase family M1 | F5U8H5_STREQ | pepN | 96 kDa |  |  | 18 (16, 14%) | 48 (38, 40%) |
| Thermophilic metalloprotease (M29) | F5U774_STREQ | pepS | 46 kDa |  |  | 1 (1, 2%) | 46 (32, 48%) |
| Peptidase family M13 | F5U9K1_STREQ | pepO | 71 kDa |  |  |  | 46 (36, 44%) |
| Oligoendopeptidase F | F5U5D6_STREQ | pepF | 63 kDa |  |  | 19 (17, 29%) | 42 (30, 38%) |
| Peptidase T | F5U486_STREQ | pepT | 45 kDa |  |  | 1 (1, 4%) | 8 (8, 20%) |
|  |  |  |  |  |  |  |  |
| Xaa-Pro dipeptidyl-peptidase | F5U4Y0_STREQ | pepX | 86 kDa |  | 1 (1, 1%) | 5 (4, 5%) | 10 (7, 9%) |
| Peptidase C1-like family | F5U669_STREQ | HMPREF9964_1078 | 47 kDa | 13 (9, 21%) | 33 (22, 49%) | 27 (21, 52%) | 20 (17, 42%) |
| M42 glutamyl aminopeptidase | F5U8R0_STREQ | HMPREF9964_2018 | 38 kDa | 19 (12, 29%) | 20 (12, 29%) | 7 (6, 17%) |  |
|  |  |  |  |  |  |  |  |
| Peptidase, C69 family | F5U6M3_STREQ | HMPREF9964_0097 | 54 kDa | 4 (3, 4%) | 1 (1, 3%) |  |  |
| Glutamyl aminopeptidase | F5U996_STREQ | pepA | 39 kDa | 4 (4, 5%) | 2 (2, 6%) | 1 (1, 4%) |  |

**Supplementary Table 5.** Relative abundances after LC-MS analysis of the phage related proteins present in the six gel fragments of the *S. agalactiae* FSL-S3 cultures that were either induced with mitomycin C or left uninduced as negative controls (Figure 6.B). The number of distinct peptides and coverage assigned for each protein are indicated in brackets. A list of all peptide sequences identified, precursor charge and m/z for each assignment, observed modification and identification score can be found in Supplemental Data 2.

|  | | |  | | |  |  |  |  |  |  |  |  |  |  |
| --- | --- | --- | --- | --- | --- | --- | --- | --- | --- | --- | --- | --- | --- | --- | --- |
|  | | |  |  | **6h induced, relative abundance** | | | | | | **Control, relative abundance** | | | | |
| **Identified Proteins** | **Accession Number** | **Molecular Weight** | | **Band A** | | | **Band B** | **Band C** | **Band D** | **Band E** | **Band A** | **Band B** | **Band C** | **Band D** | **Band E** |
| Phage-associated cell wall hydrolase | F8XZL0_STRAG | 53 kDa | | 15 (14,44%) | | |  | 6 (6,16%) | 6 (6,29%) |  | 4 (4,10%) |  | 2 (2,8%) | 2 (2,7%) |  |
| Prophage LambdaSa04, DNA polymerase | F8XZH3_STRAG | 74 kDa | | 45 (20,57%) | | | 13 (8,20%) | 4  (2,7%) |  |  | 20 (12,34%) | 19 (9,25%) | 3 (2,5%) |  |  |
| Prophage LambdaSa04, HK97 family major capsid protein | F8Y0R9_STRAG | 45 kDa | | 58 (19,80%) | | | 63 (21,79%) | 64 (9,66%) | 16 (7,45%) | 5 (3,17%) | 9 (1,23%) | 30 (11,67%) | 16 (2,45%) |  |  |
| Hypothetical phage protein | F8Y0U1_STRAG | 42 kDa | |  | | | 14 (6,31%) |  |  |  |  | 12 (7,31%) |  |  |  |
| Prophage LambdaSa04, phi13 family major tail protein | F8Y0R4_STRAG | 21 kDa | |  | | |  | 23 (5,81%) | 23 (4,81%) | 4 (1,25%) |  |  | 21 (3,75%) | 10 (3,64%) | 2 (1,16%) |
| Hypothetical phage protein | F8Y0U2_STRAG | 20 kDa | |  | | | 2 (1,16%) | 18 (12,80%) | 73 (29,91%) | 8 (4,48%) |  |  | 14 (8,52%) | 15 (9,58%) | 9 (5,53%) |
| Hypothetical phage protein | F8Y0U0_STRAG | 11 kDa | |  | | |  |  | 8 (5,70%) | 25 (15,81%) |  |  |  | 4 (2,34%) | 12 (7,79%) |
| Prophage LambdaSa04, holin | F8XZK9_STRAG | 15 kDa | |  | | |  |  | 6 (4,25%) | 15 (12,46%) |  |  |  | 2 (2,17%) | 7 (5,30%) |
| Hypothetical phage protein | F8Y0T6_STRAG | 20 kDa | |  | | |  |  | 7 (5,45%) |  |  |  |  |  |  |
| Prophage LambdaSa04, HK97 family portal protein | F8XZJ6_STRAG | 51 kDa | |  | | |  |  | 3  (0,8%) |  |  |  |  |  |  |
| Prophage LambdaSa04, head-tail adaptor | F8Y0R7_STRAG | 13 kDa | |  | | |  |  |  | 5 (4,50%) |  |  |  |  | 5 (4,55%) |


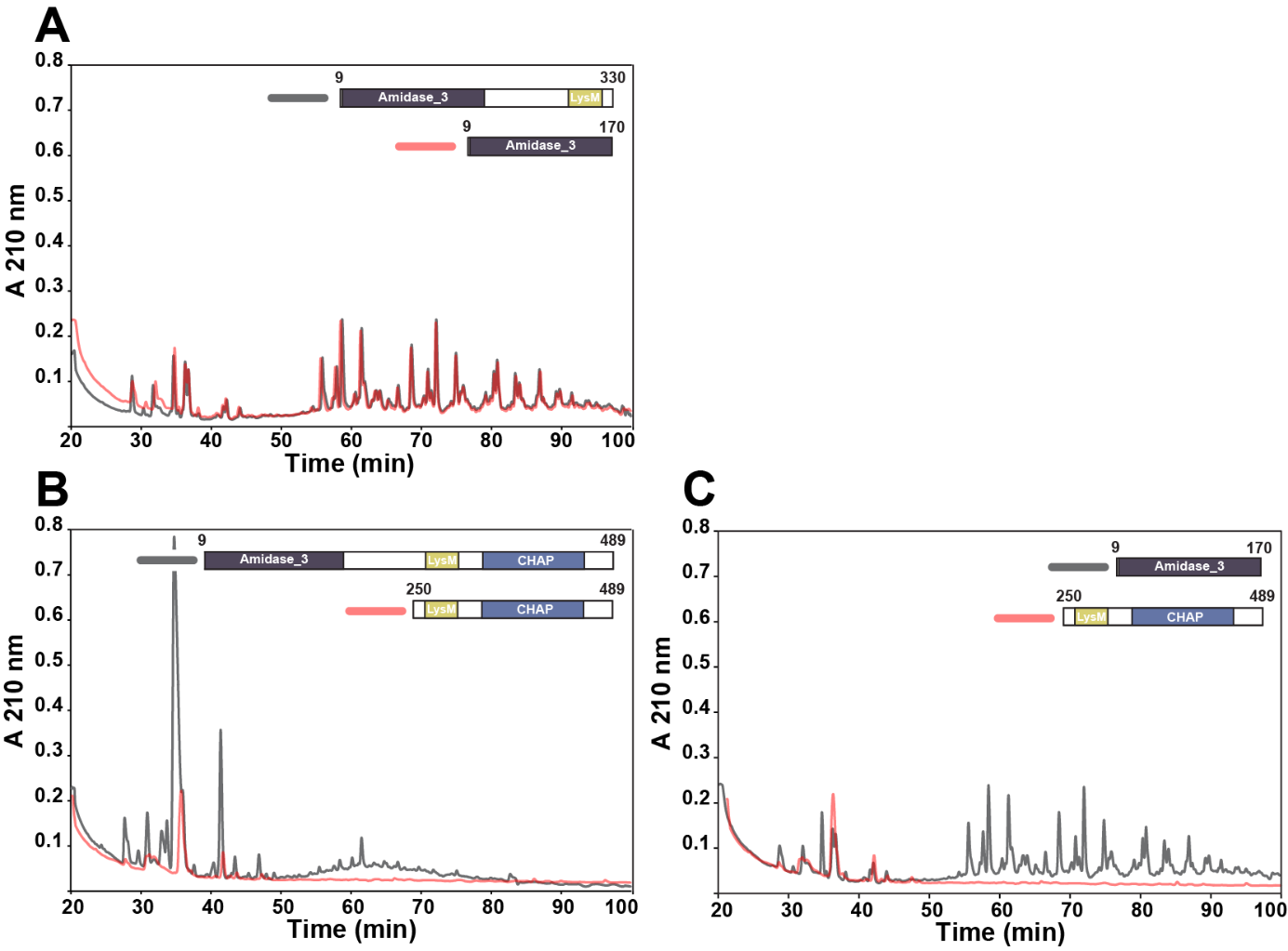


**Supplementary Fig 1. Comparison of the different chromatograms obtained from the RP-HPLC analysis of *S. dysgalactiae* peptidoglycan digested with PlySK1249 or its truncated catalytic domains.** Purified wall-peptidoglycan was digested overnight and glycans were sequentially precipitated before chromatography on a C18 Sephasil column. Equimolar concentrations (3.5 µM) of PlySK1249, Ami, Ami_LysM, and LysM_CHAP were used and analyses were repeated three times for each, yielding the same results. The chromatograms of Ami and Ami_LysM (A), PlySK1249 and LysM_CHAP (B) or Ami and LysM_CHAP (C) were superimposed for better comparison.


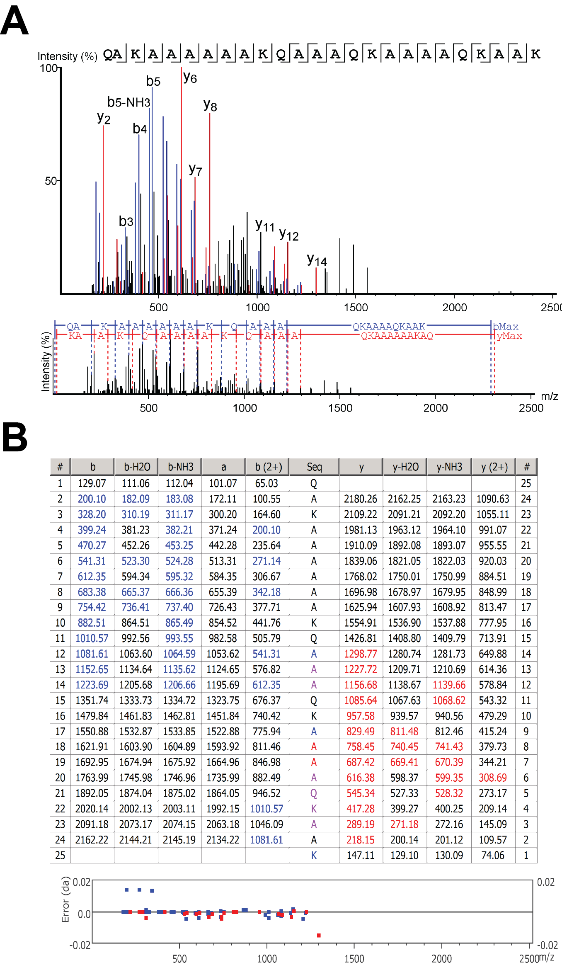


**Supplementary Fig 2: Example for the quatrimeric peptide structure sequencing.** Annotated MS/MS spectrum (A) and fragment table (B) of peptidoglycan peptide Mr 2307.313 (m/z 577.836). They were obtained by *de novo* sequencing using PEAKS 8.0. Matched b and y fragments ions are displayed in blue and red respectively in the MS/MS spectrum and the table. Residue local confidence calculated by PEAKS is shown by sequence color: blue > 60%, purple > 80%, red > 90%." The difference between the theoretical and the experimental peptide mass is 1.0 ppm."


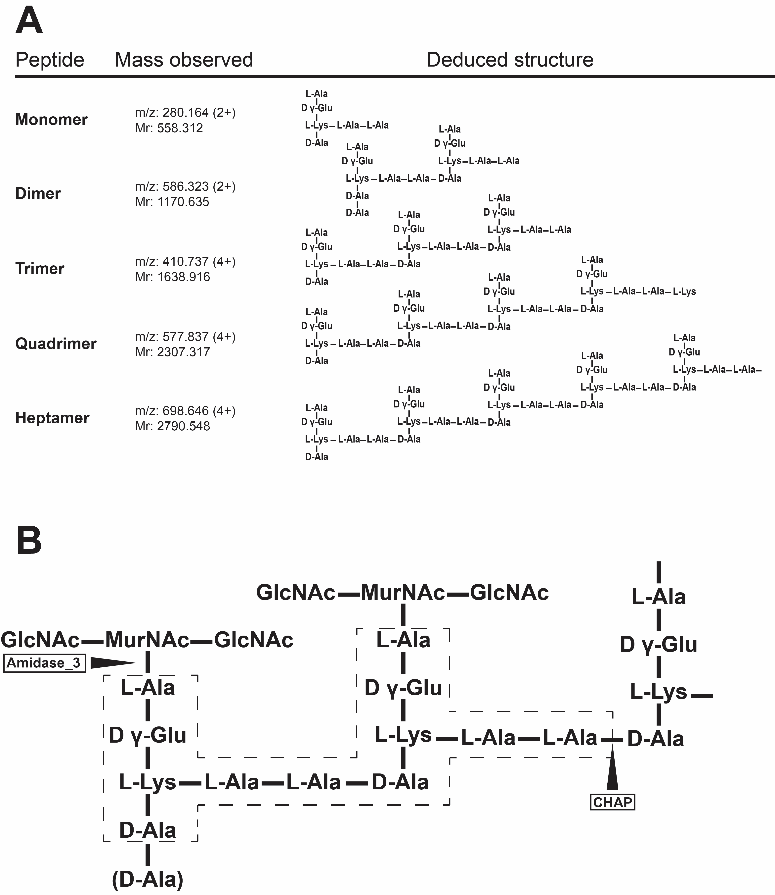


**Supplementary Fig 3. LC-MS analysis of *S. dysgalactiae* wall-peptidoglycan digested with PlysSK1249 or Ami_LysM.** A) The products of peptidoglycan digestion by the native enzyme and its truncated Ami domain were analysed by LC-MS after a 5 kDa filtration. The masses of the precursors corresponding to the dimer, trimer, quadrimer and heptamer of the AAAQKA monomer block were detected. All oligomers were detected in both samples, with the exception of the heptamers, which were only present in the amidase digestion. Polymer structures were deduced from masses after *de novo* peptide sequencing.


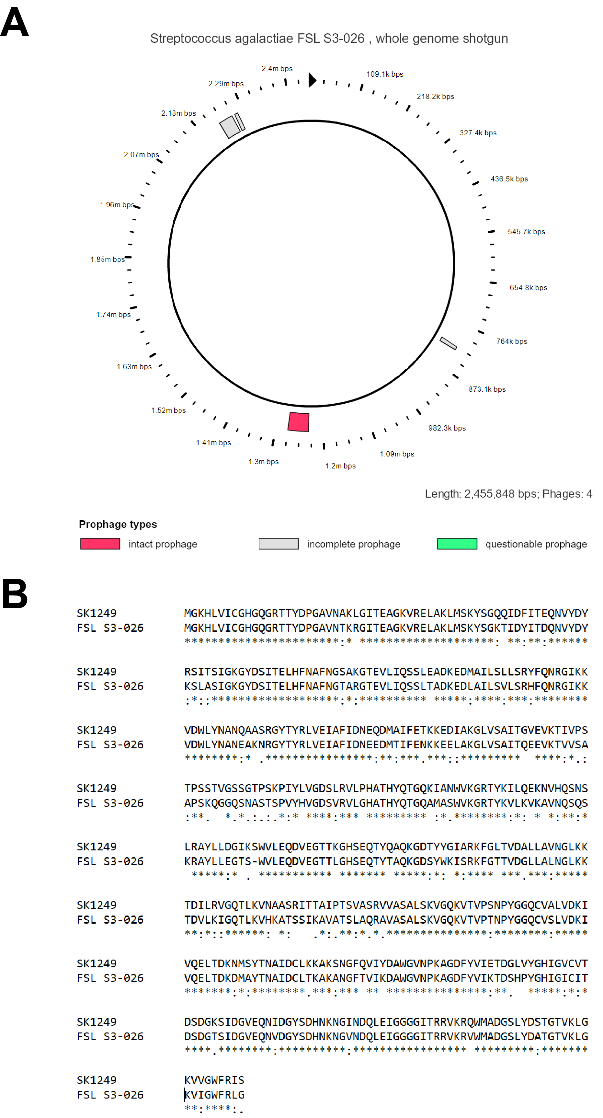


**Supplementary Fig 4: Prophage content of strain *S. agalactiae* FLS-S3-026 and alignment of its prophage endolysin compared to the PlySk1249 enzyme of strain *S. dysgalactiae* SK-1249.** A) The genome of the strain *S. agalactiae* FLS-S3-026 was analysed using PHAST and only one intact prophage was predicted. The intact prophage (PHAGE_Strept_PH10 like) harbored an endolysin sharing 78% aa homology with the PlySK1249 enzyme of strain *S. dysgalactiae* SK1249(B).


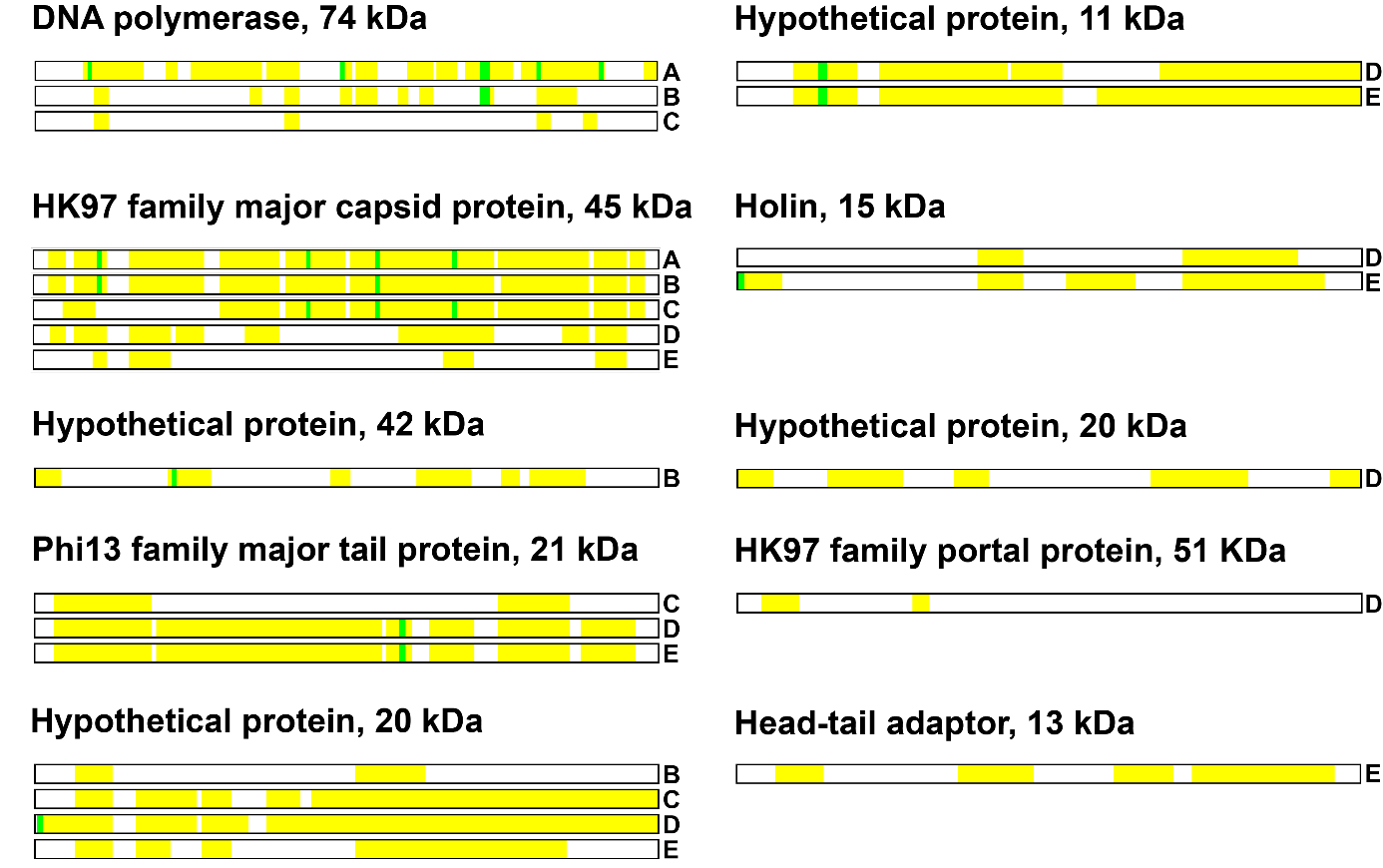


**Supplementary Fig 5: Peptide coverage of the other phage proteins detected in the induced supernatant of strain *S. agalactiae* FSL-S3.** Extracted bands from the gel of the mitomycin induced fraction (Fig. 6B) were also analysed by LC-MS for the presence of other prophage proteins. A total of 10 prophage proteins could be identified and the detected peptides that matched with the amino acid sequences are highlighted in yellow.
